# Characteristics of surveillance systems for suicide and self-harm: A scoping review

**DOI:** 10.1371/journal.pgph.0003292

**Published:** 2024-07-02

**Authors:** Aline Conceição Silva, Amanda Sarah Vanzela, Laysa Fernanda Silva Pedrollo, John Baker, José Carlos Marques de Carvalho, Carlos Alberto da Cruz Sequeira, Kelly Graziani Giacchero Vedana, José Carlos Pereira dos Santos

**Affiliations:** 1 Health Sciences Research Unit: Nursing–UICISA: E, Nursing School of Coimbra (ESEnfC), Coimbra, Portugal; 2 University of São Paulo, School of Nursing, Sao Paulo, Sao Paulo, Brazil; 3 University of São Paulo at Ribeirao Preto College of Nursing, Ribeirao Preto, Sao Paulo, Brazil; 4 School of Healthcare, University of Leeds, Leeds, United Kingdom; 5 CINTESIS—Center for Health Technology and Services Research, Porto Nursing School, Porto, Portugal; NIMHANS: National Institute of Mental Health and Neuro Sciences, INDIA

## Abstract

**Background:**

Suicide is a complex public health issue. Surveillance systems play a vital role in identifying trends and epidemiologic needs, informing public health strategies, and tailoring effective context-based suicide prevention interventions.

**Aim:**

To identify and summarise the characteristics of specific surveillance systems and general health behaviour that include data onsuicide and self-harm.

**Method:**

A scoping review following the JBI recommendations and PRISMA-ScR guidelines identified 29 relevant studies on suicide and self-harm surveillance systems. A systematic search was performed on Cinahl, Embase, Lilacs—Latin American and Caribbean Health Sciences Literature, PubMed—US National Library of Medicine, Scopus, and Google Scholar. The eligibility criteria include papers that use qualitative, quantitative or mixed methods with no restrictions on time or language. The following papers were excluded regarding euthanasia and assisted suicide, as well as papers that did not explicitly describe suicide, self-harm, and surveillance systems. Two researchers independently screened the materials for eligibility and extracted data from the included studies. Data analysis was conducted using content analysis.

**Results:**

Twenty-nine references were included, and 30 surveillance systems were identified and classified into general health behaviour surveillance (n = 15) and specific systems for suicide and self-harm (n = 15). General health behaviour systems often operate at national data collection level, collecting non-fatal data in healthcare settings, mainly emergency departments. The specific systems exhibited greater variability in terms of context, involved actors, data collection level, data collection procedures, and case classification. Limitations found by the studies pointed mostly to case definitions and data quality. Co-production, intersectoral collaboration, clear case definition criteria and data standardisation are essential to improve surveillance systems for suicide and self-harm.

**Conclusions:**

This review identified the characteristics of surveillance systems for suicide and self-harm. Monitoring and evaluation are crucial for ongoing relevance and impact on prevention efforts.

## Introduction

Suicide and self-harm are complex and multifactorial behaviours with significant health and social impacts. They are recognised as important public health issues [1, 2]. Understanding these behaviours is complex due to the involvement of multiple social, economic, political, and health aspects. Consequently, a comprehensive understanding of suicide and self-harm needs to consider these multiple dimensions, which present both possibilities and challenges that can significantly affect society and the quality of care provided to vulnerable individuals [1, 3–6].

Studies have shown that there is no clear consensus on the definition of suicidal behaviour, especially concerning self-harm. The conceptualisation of these behaviours remains unclear and dependent on the specific context and lens of analysis [5, 7]. For this study, the authors have opted to use the term “suicide”, defined as a death resulting from intentional self-harm [2, 5, 8]. On the other hand, self-harm encompasses any intentional act of self-injury or poisoning, irrespective of the apparent purpose. It is worth noting that this definition excludes repetitive or stereotypical self-injurious behaviours (behaviours without symbolic explanation or associated to neurodevelopmental disorders) [2, 8].

The available data on suicide and self-harm highlight the necessity for further studies investigating these behaviours and their impact on individual and community quality of life. In 2019, over 703.000 deaths by suicide were registered worldwide [4]. Suicide is the second leading cause of death in people aged 19–25 years [4]. In addition, data highlights that 77% of suicide occur in Low and Middle-Income Countries (LMCIs) [4, 9].

To effectively identify trends and at-risk populations, timely self-harm monitoring and registration are crucial. Improved data surveillance can facilitate the design of targeted interventions and strategies tailored to specific needs and contexts [2]. However, challenges can be encountered in this process. Despite the release of national and global data on suicide and self-harm, there are noticeable gaps in the characteristics and quality of registration, particularly regarding suicide attempts and self-harm [5, 10, 11]. The World Health Organization (WHO) highlights significant discrepancies in surveillance and data quality worldwide, with only 87 out of the 180 WHO member states providing high-quality suicide data [2, 4].

Moreover, it is worth noting that out of the 140 LMICs, only 78 have a suicide surveillance system available [8]. Many countries rely only on hospital data for information on suicide or self-harm, which may only represent part of the actual cases. This limited view could be just the tip of the iceberg, considering that an essential part of self-harm occurs within the community and would not be admitted in hospitals or specialised settings [2].

Public health surveillance systems can be defined as tools that offer indispensable data regarding the prevalence, incidence, and features of health conditions [2]. The data collected through surveillance systems play a vital role in helping government and healthcare sectors identify trends and epidemiologic needs, informing the development of public health strategies and suicide prevention interventions [2, 12–14].

The failure to collect timely data on suicide and self-harm might affect knowledge about the phenomenon, the quality of healthcare provided, and the design of public health policies and strategies for management and treatment [14–16]. Studies have shown that there is still a gap in the understanding of surveillance systems, including their characteristics, usability, and effectiveness in the context of suicide prevention on a global scale [2, 8, 14, 17, 18].

Considering the significance of strengthening suicide and self-harm surveillance systems, it is crucial to enhance and improve the understanding and care provided to people affected by suicide and self-harm. International guidelines have emphasised the need for enhanced health surveillance systems, as they have the potential to facilitate timely monitoring and rapid response and contribute to suicide prevention [2, 8, 15]. This is aligned with the Comprehensive Mental Health Action Plan (2013–2030) and the UN Sustainable Development Goals [2, 4].

Therefore, considering the relevance and identified gap regarding the characteristics and effectiveness of suicide and self-harm surveillance systems globally, this study aims to identify and summarize the characteristics of specific surveillance systems and general health behaviour that include data on suicide and self-harm. To achieve this, the following review question was elaborated: What are the characteristics of specific surveillance systems and general health behaviour that include data on suicide and self-harm?

## Materials and methods

### Study type

A scoping review following the Joana Briggs Institute (JBI) recommendations and the PRISMA-ScR (Preferred Reporting Items for Systematic Reviews and Meta-Analyses Extension for Scoping Reviews) checklist was conducted [19–22]. A protocol for this review was registered on the *Open Science Framework* [23].

### Inclusion and exclusion criteria

This review focused on studies examining suicide and self-harm surveillance systems. The inclusion criteria encompassed original studies investigating surveillance systems for suicide and self-harm, using qualitative, quantitative, or mixed methods approaches. No filters restricting publication date or language were used at this point. However, the search for grey literature was only conducted using strategies in English, Portuguese, and Spanish. When deemed relevant to the research question, studies not available online were retrieved through either a) university-affiliated Virtual Private Networks (VPNs) for access to subscribed scientific journals or b) direct contact with the studies’ authors. These measures were employed to mitigate limitations arising from a potentially limited pool of available studies.

Studies were excluded from the analysis process when: a) they discussed or focused on euthanasia and assisted suicide; b) they did not explicitly describe suicide and self-harm, limiting the understanding of the field of study and the methodological design; c) they did not delineate the characteristics of surveillance systems (e.g., studies disseminating epidemiological data collected via surveillance systems or providing general recommendations for systems); and d) they explored the reporting of suicide and self-harm in the media or to families and friends rather than the registration of cases within surveillance systems.

### Search strategy

To identify relevant studies, a comprehensive three-step approach was used (initial search of terms in databases and Mesh terms, retrieval of results from databases, and the search for results in grey literature) [19–21]. Initially, a preliminary search, in collaboration with a librarian, was conducted on PubMed (NCBI). This initial search aimed to identify relevant keywords in study titles and abstracts, enabling the formulation of the final search strategy. The identified keywords were strategically combined using Boolean operators (AND/OR/NOT) in a systematic search aligned with the research question and a mnemonic for population, concept, and context—PCC framework developed specifically for this study (S1 Table).

The final systematic search was conducted on 20^th^ June 2023, in the following databases: Cinahl, Embase, Lilacs (Latin American and Caribbean Health Sciences Literature), PubMed (US National Library of Medicine), Scopus, and WebScience. Additionally, a search was done on Google Scholar to identify grey literature. For Google Scholar and Lilacs, the authors also used terms in Portuguese and Spanish alongside English, considering the particularities of both databases. Due to its coverage, only the first 200 results were extracted from Google Scholar [24]. Terms in Portuguese and Spanish were added to the Lilacs and Google Scholar searches. References were identified and screened for eligibility (n = 2,801). To ensure a comprehensive search, two researchers independently screened the references, facilitating the identification and inclusion of additional relevant studies.

### Source of evidence screening and selection

Identified references were imported into the EndNote reference manager, and de-duplicated, before being transferred to Ryyan software for screening. To ensure a rigorous and impartial selection process, two independent researchers (AV and AS) assessed titles and abstracts against a predefined inclusion criteria [25]. Discrepancies or disagreements during the screening process were resolved through discussions between the two researchers without requiring the involvement of a third party.

### Data extraction and analysis

Two researchers (AV and AS) independently extracted data using a charting instrument based on the JBI Template Source of Evidence Details, Characteristics, and Results Extraction Instrument [19, 26]. The extraction chart included a) study details, such as characteristics, year of publication, country, context, and other pertinent information, and b) an adapted structure for collecting data on specific components. This study used components related to system characteristics (e.g., data custodians, data items, data format, data security, privacy, and confidentiality). Any discrepancies that emerged during the process were resolved upon completion.

The data were analyzed using Content Analysis [27], involving pre-analysis steps, material exploration, categorization, treatment, and interpretation of results. The data were presented based on evidence details, characterization components, and descriptions of surveillance systems. The results are presented descriptively following PRISMA-ScR guidelines, using charts, figures, and tables as necessary.

## Results

**Fig 1:**
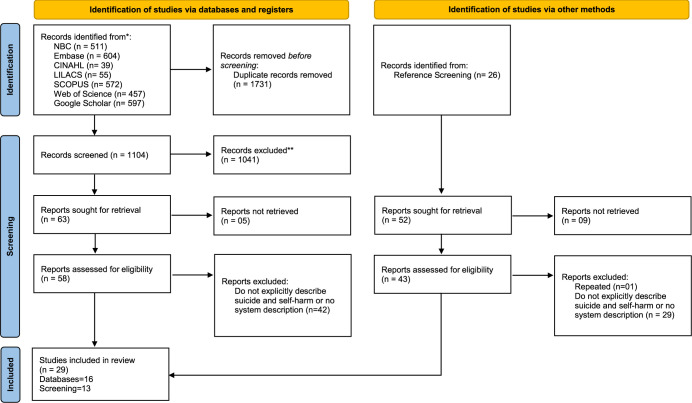
PRISMA 2020 flow diagram for the study selection process.

A total of 2,835 references were identified across the databases. After duplicate removal and screening of abstracts and titles, 63 were selected for full-text screening. Five of these could not be retrieved, and 42 were excluded for not providing detail about the surveillance system and/or specifying the behaviour of interest (suicide or self-harm). Citation screening of included papers (n = 16) identified an additional 13 papers for inclusion (Fig 1).

Reports excluded:Repeated (n=01)Do not explicitly describe suicide and self-harm or no system description (n = 29)The included references (n = 29) were published between 1993 and 2023 and were scientific articles (n = 20), followed by guidelines (n = 8) and a master’s dissertation. Among the included scientific articles (n = 20), 16 were qualitative, three were quantitative, and one used a mixed-method approach. The materials were primarily produced in North America (n = 10), followed by South America (n = 7), Asia (n = 3), Europe (n = 4), Oceania (n = 2), Central America (n = 2), and Africa (n = 1), and were written in English (n = 20), Spanish (n = 5), and Portuguese (n = 4). The primary objective of the materials was to describe or evaluate surveillance systems (Table 1).

**Table 1:** Description of included references for surveillance systems for suicide and self-harm.

N	Reference	Year	Country	Study design	System
1	Brockie et al [28]	2023	United States	Qualitative	Celebrating Life (CL)
2	Marzano et al [29]	2023	United Kingdom	Qualitative	The police led RTSS system (RTSS)
3	COMISCA [30]	2022	Central America	N/A	Central American Integration System (SICA)
4	Benson et al [26]	2022	United Kingdom	Qualitative	Coronial Suspected Suicide Data Sharing Service (CDS), Interim Queensland Suicide Register (iQSR), Victorian Suicide Register (VSR), Thames Valley Police Real-Time Suicide Surveillance (TV-RT-SSS), Suicide and Self-Harm Observatory (SSHO)
5	Ehlman et al [31]	2021	United States	Mixed Methods	National Electronic Injury Surveillance System—All Injury Program (NEISS-AIP)
6	Vijayakumar et al [32]	2020	India	Quantitative	Suicide Prevention and Implementation Research Initiative (SPIRIT)
7	Lubman et al [33]	2020	Australia	Qualitative	National Ambulance Surveillance System (NASS)
8	Ministério da Saúde [15]	2019	Brazil	N/A	Notification of Diseases Information System (SINAN)
9	Ministerio de Salud Pública [34]	2018	Guatemala	N/A	National Epidemiological Surveillance System
10	Secretaria de Salud [35]	2018	Colombia	N/A	National Public Health Surveillance System (SIVIGILA)
11	Sutherland et al [18]	2017	Australia	Qualitative	Victorian Suicide Register (VSR)
12	Ministério da Saúde [36]	2016	Brazil	N/A	Surveillance System for Violence and Accidents (VIVA)
13	Hoffmire et al [37]	2016	United States	Quantitative	Suicide Prevention Applications Network (SPAN)
14	Blair et al [38]	2016	United States	Qualitative	National Violent Death Reporting System (NVDRS)
15	Ribeiro [39]	2016	Brazil	Quantitative	Notification of Diseases Information System (SINAN), Mortality Information System (SIM)
16	Williams [40]	2015	England	Qualitative	Self-Harm Surveillance Register (SHSR)
17	Ministerio de Salud y Protección Social [41]	2014	Colombia	N/A	Epidemiological Surveillance System for Suicidal Behavior (SISVECOS)
18	Cwik et al [42]	2014	United States	Qualitative	Apache Surveillance System (ASS)
19	Ministerio de Salud [43]	2013	Argentina	N/A	Injury Surveillance System (SIVILE)
20	Montevalian et al [44]	2011	Iran	Qualitative	Injury Surveillance System (ISS)
21	Steenkamp et al [45]	2006	United States	Qualitative	National Violent Death Reporting System (NVDRS)
22	Chiang et al [46]	2006	Taiwan	Qualitative	National Suicide Surveillance System (NSSS)
23	Paulozzi et al [47]	2004	United States	Qualitative	National Violent Death Reporting System (NVDRS)
24	Ward et al [48]	2002	Jamaica	Qualitative	Jamaica InjurySurveillance System (JISS)
25	Arscott-Mills [49]	2002	Jamaica	Qualitative	Accident and emergency statistical report (A&ESR): Patient Administration System/Jamaica Injury Surveillance System (PAS/JISS)
26	Ministério da Saúde [50]	2001	Brazil	N/A	Mortality Information System (SIM)
27	Butchart et al [51]	2001	South Africa	Qualitative	National Non-Natural Mortality Surveillance System (NMSS)
28	Mackenzie et al [52]	1999	Canada	Qualitative	Canadian Hospitals Injury Reporting and Prevention Program (CHIRPP)
29	Birkhead et al [53]	1993	Georgia	Quantitative	Emergency Department Surveillance System (EDSS)

The systems were categorised into either a) general health behaviour surveillance systems that included suicide and self-harm (n = 15) or b) specific surveillance systems for suicide and self-harm (n = 15).

### Surveillance systems for general behaviours (including suicide and self-harm)

Fifteen surveillance systems covering general behaviours, including suicide and self-harm, were identified. Most of these systems addressed health-related behaviours related to injuries broadly [31, 43, 44, 48, 49, 52], injuries due to violence [36], health-related incidents [15, 36, 39], poisoning, external causes, suicide [34], mental health [30, 33], mortality [39, 50], mortality due to violence [45], or non-natural causes [51]. These systems predominantly operated at a national level for data collection [15, 31, 33, 34, 36, 39, 43, 44, 49, 51], primarily in a healthcare context [30, 31, 33, 39, 41, 43, 44, 48, 49, 50, 52], and specifically within emergency departments [30, 44, 48, 49, 52].

References that focused on surveillance systems in emergency departments highlighted challenges in defining precise variables for recording, data incompleteness [31, 44, 49, 52], or incorrect data entry [49]. Additional challenges included: poor hospital engagement in the surveillance system’s performance improvement [31], a lack of training and capacity building for human resources [49], the absence of records for injured individuals referred to other services [44], and limited dissemination of data [31, 44, 49] (S2 Table). In this context, the importance of coding for case recording, the use of protocols and reference materials for consultation and training, and team awareness regarding the importance and necessity of data recording were emphasised [48].

The systems focused on non-fatal outcomes [15, 31, 33, 36, 39, 48, 49, 52] or both fatal and non-fatal outcomes [30, 36, 40, 43, 44] and provided different definitions of the behaviour based on the intentionality of the acts (S1 Table). In most cases, socio-demographic, psychosocial, and psychiatric information was collected, often through clinical assessments conducted by medical professionals (Table 2). Most systems used the International Classification of Diseases—ICD [15, 34, 36, 39, 45, 50, 51] (Table 2).

**Table 2:** Main characteristics of surveillance systems for general health behaviours (including suicide and self-harm).

System	Level	Context	Health Behaviour	Concept	Classification
SICA	International	Health	Mental Health	Attempt, suicide	Various
SINAVE	National	Health	Poisoning, external causes, suicide	Attempt, suicide	ICD
SIVILE	National	Health	Injury	Attempt, suicide	System Code
IS System	National	Health	Injury	Attempt, suicide	Record
SINAN	National	Intersectoral	Illness	Attempt	ICD
SIM	National	Health	Mortality	Suicide	ICD
VIVA	National	Intersectoral	Violence	Attempt, suicide	ICD
NASS	National	Health	Mental health	Self-harm	ePCR
NMSS	National	Medico-legal	Non-natural mortality	Suicide	ICD
NEISS-AIP	National	Health	Injury	Nonfatal SDV	Record
A&ESR	National	Health	Injury	Attempt	Record
*PAS/JISS	National	Health	Injury	Attempt	-
NVDRS	National*	Medico-legal	Violence Mortality	Suicide	ICD
CHIRPP	Provincial	Health	Injury	Self-harm	System Code
JISS	Regional	Health	Injury	Attempt	System Code

*Systems presented on the same included material. ICD: International Classification of Diseases. Intersectorial: Involves different social sectors (e.g., health, social assistance, security, among others). ePCR: electronic Patient Care Record. Medico-legal: Involves legal and judicial systems in determining the case. SDV: self-directed violence.

Three mortality surveillance systems were identified, including one initiative in the United States that collected data about violent deaths from primary systems [38, 45, 47]. A Brazilian initiative collected data from death certificates for mortality surveillance in the country [39, 50]. Both systems used the ICD as the classification criterion [38, 39, 50] and publicly disseminated data through websites and reports (DataSUS) [39, 50], annual webinar (CDC) and written reports [39]. Additionally, a South African initiative collected data from medicolegal investigations of non-natural deaths [51]. These highlighted the challenges in using different case definitions [39], coding and data entry [39, 49, 51], inconsistency in information sources [39], organisational acceptance difficulties, funding, and system continuity [51]. The papers on emergency department and mortality systems pointed out the challenge of assessing data sensitivity [49, 51].

Furthermore, a Brazilian surveillance system for violence and accidents [36] was identified, gathering suicide attempt data nationally with two components: continuous surveillance based on information systems [15, 36, 39] and sentinel surveillance with epidemiological surveys. Continuous surveillance was conducted in an intersectoral context involving all three levels of the healthcare system (primary, secondary, and tertiary), social assistance, education, child protection councils (permanent and autonomous bodies responsible for ensuring children’s and adolescents’ rights compliance), rights councils, protection councils, justice councils, as well as governmental, non-governmental, and private sector organisations. Also, an international system in Central America and the Dominican Republic collected data about mental in primary care for planning interventions at different levels (national level in Central American and regional level in Dominican Republic) [30] (S2 Table).

### Specific suicide and self-harm surveillance systems

Fifteen references described systems specifically for suicide and/or self-harm surveillance. These systems expanded the identification of cases with non-fatal outcomes (ideation, threat, nonsuicidal self-injury—NSSI, attempt) and had varying levels of data collection (state-based, district, regional, and local). Most of these systems were situated in a healthcare context [35, 37, 41, 53], especially in emergency departments [40, 41, 53], and one system for war veterans, a perceived high-risk group in the United States [37]. Additionally, there were mortality systems based on coroner’s investigations [26, 29]. Most systems developed their own classification criteria [26, 28, 32, 37, 41, 42, 46], with a notable emphasis on using Rosemberg et al.’s (1988) criteria (operational criteria to assist coroners and medical examiners in determining suicide) and consensus procedures. In cases of suspected suicide, most systems used coronial investigations (Table 3 and S3 Table).

**Table 3:** Main characteristics of specific suicide and self-harm surveillance systems.

System	Local	Level	Context	Conception	Classification
SIVIGILA	Colombia	National	Health	Attempt	Own classification
NSSS	Taiwan	National	Intersectoral	Attempt	Own classification
*CDS	New Zealand	National	Coroner	Suicide	Coronial investigation
SPAN^4^	EUA	National	Health	Ideation, attempt, death	Suicide Behaviour Report (EBRs)
RTSS	United Kingdom	National	Coroners and police	Suicide	Police and coronial investigation
*iQSR	Queensland	State	Coroner	Suspected suicide	Coronial investigation
*VSR	Victoria	State	Coroner	Suspected suicide	Coronial investigation
SISVECOS	Bogotá	District	Health	Ideation, threat, attempt	Own classification
*TV-RT-SSS	Thames Valley	Regional	Police	Suspected suicide	Police and Coronial investigation
*SSHO	Count Cork	Regional	Research	Suspected suicide	Rosenberg criteria
SPIRIT	Mehsana	Regional	Community	Attempt, suicide	Case Report Form
EDSS	Cobb County	Regional	Health	Ideation, attempt	ICD + Rosenberg criteria
ASS	Fort Apache Indian Reserve	Local	Community	Ideation, attempt, suicide, NSSI	Consensus procedure
SHSR	Southwest England	Local	Health	Self-harm	ED’s risk assessment tool
CL	Fort Peck Reserve	Local	Community	Ideation, attempt, suicide	Intake and case management forms

*Systems presented in the same included material. Rosemberg criteria: Rosenberg et al., 1988. Gen 19: Sudden death Form (Gen 19). ICD: International Classification of Diseases.

Systems using coronial investigations [26, 29] collected cases labelled as "suspected suicide", "open verdict" or "identity not yet confirmed," primarily from coroners who conducted investigations based on various sources of information (medical records, police reports, witness statements, family reports). Cases were reviewed and classified after the coronial investigation or inquiry [26]. The coronial systems require nine months to two years to complete an investigation process. In this sense, the real-time suicide surveillance described in the study contributes to the sharing and analysis of data (with different timelines among the systems) to aid in the planning and implementation of immediate suicide prevention actions.

This data was used for surveillance and local responses to suspected suicides (CDS), early responses to groups (clusters or contagion) (CDS, TV-RT-SSS), investigation of specific clusters or subgroups (iQSR), and facilitating early responses to suicides, associated contagions, and grieving communities (SSHO). Subsequently, the data is reviewed based on coroners’ verdicts, but the emphasis of the real-time surveillance system is on the potential for early prevention strategies. The systems collectively reported high sensitivity and satisfaction, serving as a model suitable for replication in other locations [26]. Studies also highlighted the use of police-led systems for data collection, as law enforcement is primarily called in cases of suspected suicides [29]. However, concerns were raised regarding the need for education and destigmatisation and concerns about police involvement in mental health actions [26].

A national integrated health system, including data from hospitals, fire departments, social services, and education systems, was described in Tawan [46]. This system aimed to identify suicide attempts and refer individuals to mental health or social services, support families (high-risk groups), and provide data for tailoring prevention strategies. However, reluctance or lack of motivation to record cases, the need for feedback and organisational incentives, alternative recording channels, and administrative support were identified as challenges [46].

In community initiatives, particularly those areas with diverse socio-cultural backgrounds (indigenous or remote communities), integration of different social resources (medical, school, and social service personnel, first responders, religious leaders, family members, and peers) was emphasised [28]. Data collection in community systems also extended beyond socio-demographic, psychosocial, and psychiatric characteristics to include cultural aspects (e.g., access to housing and traditional ceremonies) [28, 42].

For example, Celebrating Life, a community-based surveillance in the Fort Apache Indian Reservation region (on the border of New Mexico and Arizona, United States), collecteds data from individuals, schools, and other social sectors through admission forms. These forms were submitted to the Celebrating Life team, which conducted follow-up visits, risk identification, and monitoring. In cases of suicide, the suicide death form was completed by the system’s responsible team. Event classification was based on declared intent, congruence of method with intent, method lethality, reported behaviour function, and simultaneous substance use. Behaviours could also be confirmed by accessing other information sources (police reports, IHS medical records, local providers, and first responders) and through consensus procedures within the team [42].

An implementation study of a suicide surveillance system in 124 villages in Mehsana, North Gujarat, India, utilized data triangulation from key informants in the community, health records, and police records. The findings from triangulating data collected through community sources, health services, and police records revealed a noteworthy number of cases identified by the community that had not been documented in health and/or police systems [32].

Studies on community-based surveillance systems highlighted collaborative development involving identifying and engaging partners, available active resources, and recognizing cultural assets such as traditional individuals, natural helpers, ceremonies, and elders familiar with traditions and language [28]. Sustainability factors included community involvement, funding, organisational capacity, recognition, communication, partnerships with clear expectations, assigned responsibilities, actions aligned with partner capacity and policies, and evaluation [28].

## Discussion

This review revealed two categories of surveillance systems: general health behaviour surveillance systems and specific suicide and self-harm surveillance systems. The general health behaviour systems, which included self-harm and suicide, mainly operated nationally within a healthcare context. They focused on non-fatal outcomes and primarily collected data in emergency departments. The specific suicide and self-harm surveillance systems included systems with varying levels of data collection, such as state-based, district, regional, and local. In these systems, a variation of contexts was also identified, with the majority in health and coronial investigation contexts but with the inclusion of other contexts such as police records, community, and research.

The scientific literature highlights the prevalence of surveillance systems focused on identifying suicides and suicide attempts [8], particularly in emergency services [8, 13]. However, the results of this study emphasize initiatives in different social contexts and particularly in addressing non-fatal outcomes. In 2022, following a series of initiatives for suicide prevention, the WHO released a manual dedicated to guiding data collection on suicide and self-harm in the community [2]. In this sense, the initiative reinforces the importance of surveillance of behaviours in the community, and comprehensive community surveillance systems can contribute to the design of suicide prevention programs and national strategies that are more representative and appropriate to the socio-cultural needs of the population [2].

Another important result was linked to the real-time dissemination of data related to suicide and self-harm surveillance. The real-time data dissemination enabled the development of local and early responses, especially for mitigating the effects on the community by investigating clusters, contagion effects, and postvention care [26]. This also underscores the discussion about surveillance systems for non-fatal outcomes to enable early identification, follow-up in the healthcare network, and suicide prevention.

An important counterpoint was the variety of terms, definitions, and classifications for suicide and self-harm. The lack of international consensus on terminologies and definitions impacts on understanding data on these phenomena [17, 54], especially in non-fatal outcomes where there are no systematic and routine data reports [54]. Regarding case classification, studies highlight the low sensitivity of ICD target codes, especially for behaviors where intentionality is not well defined [55, 56]. This issue may also be linked to the results of this study, where most specific systems for non-fatal outcomes used their own classifications for case coding.

The classification of cases and coding directly influence institutional and professional data entry processes into systems and have a direct impact on the surveillance of behaviours, such as determining cases with undetermined intent. This process consequently influences suicide rates, investment, and the formulation of public prevention policies [57]. From this perspective, the results also highlight significant challenges such as lack of training and professional development for surveillance, overload and the need for incentives, motivation, and professional recognition.

Finally, it is worth noting that global surveillance of suicide and self-harm faces several challenges, particularly when considering the criminalization of such behaviours. Among 52 countries, suicide is considered a crime punishable by criminal prosecution in 25, while in 27, the legal frameworks are undetermined. The criminalization of these behaviours directly impacts suicide prevention strategies, including surveillance and public health policies [58]

Approaches to surveillance systems have the potential to be expanded globally [8, 13]. Official suicide rates have been used to track trends and monitor the impact of changes in legislation, treatment policies, and social changes Therefore, the importance of investing in and expanding surveillance for public health is highlighted in the development and evaluation of strategies for care and suicide and self-harm prevention [17, 54].

Limitations of this study should be considered. First, the identification of systems was confined to the criteria outlined in the design and selection of this study. In this sense, it does not represent the characteristics of all available systems. Second, the identification focused on descriptive features, necessitating the assessment of other operational and practical characteristics. Third, no efforts were made to update information on websites or directly contact the responsible entities for surveillance systems. Fourth, the absence of searches in repositories or other sources of grey literature. And fifth, the exclusion of systems specifically utilizing machine learning in the surveillance of suicide and self-harm.

## Conclusion

This scoping review provides an overview of the characteristics of suicide and self-harm surveillance systems, highlighting variations in definitions, data collection settings, and challenges these systems face. The findings highlight the importance of intersectoral collaboration, the development of clear criteria for case classification, and efforts to address data quality issues in suicide and self-harm surveillance systems.

Understanding the characteristics, possibilities and challenges of surveillance systems is essential for improving self-harm and suicide data and, therefore, prevention efforts. Future research should focus on addressing the identified challenges and promoting the standardisation of definitions and data collection methods to enhance the effectiveness of these surveillance systems. Additionally, ongoing monitoring and evaluation of these systems are necessary to ensure their continued relevance and impact on suicide and self-harm prevention efforts.

## Supporting information

S1 Table Search strategy based on the mnemonic PCC (mnemonic for population, concept and context). (DOCX)

S2 Table Synthesis of the characteristics of general surveillance systems (includes suicide and self-harm). (DOCX)

S3 Table Synthesis of the characteristics of specific surveillance systems for suicide and self-harm. (DOCX)

## References

[1] Yip PSF, Zheng Y. The Impact of suicide on life expectancy. Crisis. 2021; 42(2):107–113. doi: 10.1027/0227-5910/a000695 32431197

[2] Organization WH. Training manual for surveillance of suicide and self-harm in communities via key informants. World Health Organization. 2022; 1–65. https://apps.who.int/iris/handle/10665/365481.

[3] Castelpietra G, Knudsen AKS, Agardh EE, Armocida B, Beghi M, Iburg KM, et al. The burden of mental disorders, substance use disorders and self-harm among young people in Europe, 1990–2019: findings from the Global Burden of Disease Study 2019. Lancet Public Health. 2022; 16:100341. doi: 10.1016/j.lanepe.2022.100341 35392452 PMC8980870

[4] Organization WH. Comprehensive mental health action plan 2013–2030. World Health Organization. 2021. https://apps.who.int/iris/handle/10665/345301.

[5] De Leo D, Zammarrelli J, Viecelli Giannotti A, Donna S, Bertini S, Santini A, et al. Notification of Unexpected, Violent and Traumatic Death: A Systematic Review. Front Psychol. 2020; 24;11:2229. doi: 10.3389/fpsyg.2020.02229 33101106 PMC7546769

[6] Gibson R, Carson J, Houghton T. Stigma towards non-suicidal self-harm: evaluating a brief educational intervention. Br J Nurs. 2019; 28(5):307–312. doi: 10.12968/bjon.2019.28.5.307 30907659

[7] Wilson E, Ougrin D. Commentary: Defining self-harm: how inconsistencies in language persist–a commentary/reflection on Ward and Curran (2021). Child Adolesc Ment Health. 2021; 26(4):372–374. doi: 10.1111/camh.12502 34414651

[8] Organization WH. Practice manual for establishing and maintaining surveillance systems for suicide attempts and self-harm. World Health Organization. 2016. https://apps.who.int/iris/handle/10665/208895.

[9] Lim KS, Wong CH, McIntyre RS, Wang J, Zhang Z, Tran BX et al. Global lifetime and 12-month prevalence of suicidal behavior, deliberate self-harm and non-suicidal self-injury in children and adolescents between 1989 and 2018: a meta-analysis. Int J Environ Res Public Health. 2019; 16(22):4581. doi: 10.3390/ijerph16224581 31752375 PMC6888476

[10] Bentley KH, Maimone JS, Kilbury EN, Tate MS, Wisniewski H, Levine MT, et al. Practices for monitoring and responding to incoming data on self-injurious thoughts and behaviours in intensive longitudinal studies: a systematic review. Clinical psychology review. 2021; 90:102098. 10.1016/j.cpr.2021.102098. 34763126 PMC8663717

[11] Silva DA, Marcolan JF. Suicide attempt and death by suicide in Brazil: an epidemiological analysis. Medicina. 2021; 54(4):e181793. 10.11606/issn.2176-7262.rmrp.2021.181793. PMC8939476 35110169

[12] Haghiri H, Rabiei R, Hosseini A, Moghaddasi H, Asadi F. Notifiable Diseases surveillance system with a data architecture approach: a systematic review. Acta Inform Med. 2019; 27(4):268–277. doi: 10.5455/aim.2019.27.268-277 32055095 PMC7004293

[13] Ikeda R, Hedegaard H, Bossarte R, Crosby AE, Hanzlick R, Roesler J, et al. Improving national data systems for surveillance of suiciderelated events. Am J Prev Med. 2014; 47(3):122–129. doi: 10.1016/j.amepre.2014.05.026 25145729 PMC4959537

[14] Marcolan JF. For a public policy of surveillance of suicidal behavior. Rev Bras Enferm. 2018; 71(5):2343–7. doi: 10.1590/0034-7167-2018-0256 30365803

[15] Brazil. Caderno de análise: roteiro para uso do sinan net, análise da qualidade da base de dados e cálculo de indicadores epidemiológicos e operacionais. MS. 2019; 1:1–75. http://portalsinan.saude.gov.br/images/documentos/Agravos/Violencia/CADERNO_ANALISE_SINAN_Marco_2019_V1.pdf. Accessed 26 May 2023.

[16] Pinheiro TP, Warmling D, Coelho EBS. Characterisation of suicide attempts and self-harm by adolescents and adults notified in Santa Catarina, Brazil, 2014–2018. RESS. 2021; 30(4):e2021337. 10.1590/S1679-49742021000400026. 34878004

[17] De Leo D, Goodfellow B, Silverman M, Berman A, Mann J, Arensman E, et al. International study of definitions of English-language terms for suicidal behaviours: a survey exploring preferred terminology. BMJ open. 2021; 11(2):e043409. doi: 10.1136/bmjopen-2020-043409 33563622 PMC7875264

[18] Sutherland G, Milner A, Dwyer J, Bugeja L, Woodward A, Robinson J, et al. Implementation and evaluation of the Victorian suicide register. Aust N Z J Public Health. 2018; 42(3):296–302. doi: 10.1111/1753-6405.12725 29044826

[19] Peters M, Godfrey C, McInerney P, Munn Z, Tricco A, Khalil H, et al. Scoping Reviews. In JBI Manual for evidence synthesis; 2020 edn. Adelaide, Australia. 2020. https://jbi-global-wiki.refined.site/space/MANUAL. 10.11124/JBIES-20-00167 33038124

[20] Peters MD, Godfrey CM, Khalil H, McInerney P, Parker D, Soares CB. Guidance for conducting systematic scoping reviews. International journal of evidence-based healthcare. 2015; 13(3), 141–146. doi: 10.1097/XEB.0000000000000050 26134548

[21] Khalil H, Peters MD, Tricco AC, Pollock D, Alexander L, McInerney P, et al. Conducting high quality scoping reviews-challenges and solutions. Journal of clinical epidemiology. 2021;130, 156–160. doi: 10.1016/j.jclinepi.2020.10.009 33122034

[22] Tricco AC, Lillie E, Zarin W, O’Brien KK, Colquhoun H, Levac D et al. PRISMA extension for scoping reviews (PRISMA-ScR): checklist and explanation. Ann Intern Med. 2018; 169(7):467–473. doi: 10.7326/M18-0850 30178033

[23] Silva AC, Vanzela AS, Pedrollo LFS et al. Surveillance systems for self-harm and suicide in healthcare: a scoping review. 2023. 10.17605/OSF.IO/D6HXA.

[24] Lefebvre C, Glanville J, Briscoe S, Featherstone R, Littlewood A, Marshall C, et al. Cochrane Handbook for Systematic Reviews of Interventions Version 6.3 (updated February 2022). Cochrane, 2022.

[25] Mendes KDS, Silveira RCCP, Galvão CM. Use of the bibliographic reference manager in the selection of primary studies in integrative reviews. Texto Contexto Enferm [Internet]. 2019; 28:e20170204. 10.1590/1980-265X-TCE-2017-0204.

[26] Benson R, Rigby J, Brunsdon C, Corcoran P, Dodd P, Ryan M et al. Real-time suicide surveillance: comparison of international surveillance systems and recommended best practice. Arch Suicide Res. 2022; 1–27. doi: 10.1080/13811118.2022.2131489 36237124

[27] Krippendorff K. (2004). Content Analysis: An Introduction to Its Methodology (2nd ed.) Thousand Oaks, CA: Sage Publications.

[28] Brockie T, Decker E, Barlow A, Cwik M, Ricker A, Aguilar T, et al. Planning for implementation and sustainability of a community-based suicide surveillance system in a Native American community. Implement Scis. 2023; 4(1). 10.1186/s43058-022-00376-1. PMC9814428 36600290

[29] Marzano L, Norman H, Sohal B, Hawton K, Mann R. Police-led real-time surveillance system for suspected suicides in Great Britain. BMJ mental health. 2023; 26(1):e300643. doi: 10.1136/bmjment-2022-300643 37085285 PMC10124228

[30] Comisca. Consejo de ministros de salud de centroamérica y república dominicana. resolución comisca. relativa al v foro intersectorial regional para la salud de centroamérica y república dominicana. uniendo fuerzas por la salud mental. 2018. https://www.sica.int/documentos/resolucion-comisca-03-2022-relativa-al-v-foro-intersectorial-regional-para-la-salud-de-centroamerica-y-republica-dominicana-uniendo-fuerzas-por-la-salud-mental_1_131807.html. Accessed 15 Jul 2023.

[31] Ehlman DC, Haileyesus T, Lee R, Ballesteros MF, Yard E et al. Evaluation of the National electronic injury surveillance system—all injury program’s self-directed violence data, United States, 2018. J Safety Res. 2021; 76:327–331. doi: 10.1016/j.jsr.2020.12.002 33653565 PMC8040093

[32] Vijayakumar L, Pathare S, Jain N, Nardodkar R, Pandit D, Krishnamoorthy S, et al. Implementation of a comprehensive surveillance system for recording suicides and attempted suicides in rural. India BMJ Open. 2020; 10:e038636. 10.1136/bmjopen-2020-038636. PMC7654119 33168552

[33] Lubman DI, Heilbronn C, Ogeil RP, Killian JJ, Matthews S, Smith K, et al. National ambulance surveillance system: a novel method using coded australian ambulance clinical records to monitor self-harm and mental health-related morbidity. PloS one. 2020; 15(7):e0236344. doi: 10.1371/journal.pone.0236344 32735559 PMC7394421

[34] Guatemala. Protocolo del sistema nacional de vigilancia epidemiológica: intoxicaciones por plaguicidas, lesiones de causa externa y conducta suicida. Dep Epidem. 2018; 10:1–37. https://epidemiologia.mspas.gob.gt/phocadownload/userupload/protocolo-de-vigilancia/vigente/10.pdf.

[35] Secretaria de Salud. Manual del usuario del aplicativo web del sistema de vigilancia epidemiologica de vigilância epidemiológica de conductas suicidas sisvecos-sivigila d.c. 2018.

[36] Brazil. Viva: instrutivo de notificação de violência interpessoal e autoprovocada. MS DANT. 2016; 2:1–92. https://bvsms.saude.gov.br/bvs/publicacoes/viva_instrutivo_violencia_interpessoal_autoprovocada_2ed.pdf. Accessed 10 May 2023.

[37] Hoffmire C, Stephens B, Morley S, Thompson C, Kemp J, Bossarte RM. VA suicide prevention applications network: a national health care system-based suicide event tracking system. Public Health Rep. 2016; 131(6):816–821. doi: 10.1177/0033354916670133 28123228 PMC5230828

[38] Blair JM, Fowler KA, Jack SP, Crosby AE. The national violent death reporting system: overview and future directions. Inj Prev. 2016; 22(1):6–11. doi: 10.1136/injuryprev-2015-041819 26718549 PMC6049659

[39] Ribeiro NM. Information Systems analysis in health SIM and SINAN toward suicide in the city of Uberaba/MG. Dissertation, Universidade Federal do Triângulo Mineiro. 2016. http://bdtd.uftm.edu.br/handle/tede/240.

[40] Williams S. Establishing a self-harm surveillance register to improve care in a general hospital. Br J Ment Health Nurs. 2015; 4:20–5. 10.12968/bjmh.2015.4.1.20.

[41] Colombia. Protocolo de vigilancia em Salud Pública: intento de suicídio. MPS. 2014; 1–19. https://www.minsalud.gov.co/sites/rid/Lists/BibliotecaDigital/RIDE/IA/INS/protocolo-vigilancia-intento-suicidio.pdf. Accessed 07 Jul 2023.

[42] Cwik MF, Barlow A, Goklish N, Larzelere-Hinton F, Tingey L, Craig M, et al. Community-based surveillance and case management for suicide prevention: an American Indian tribally initiated system. Am J Public Health. 2014; 104(3): 18–23. doi: 10.2105/AJPH.2014.301872 24754618 PMC4035881

[43] Argentina. Instructivo para el registro de datos, en unidades centinelas del sistema de vigilancia de lesiones, SIVILE. MS Argentina. 2013; 1–44. https://bancos.salud.gob.ar/sites/default/files/2020-01/instructivo-registro-datos-en-unid-centinelas-sist-vigilancia-lesiones.pdf.

[44] Montevalian SA, Haddadi M, Akbari H, Khorramirouz R, Saadat S, Tehrani A, et al. Strengthening injury surveillance system in Iran. Chin J Traumatol. 2011; 14(6):348–353. 22152138

[45] Steenkamp M, Frazier L, Lipskiy N, Deberry M, Thomas S, Barker L, et al. The National violent death reporting system: an exciting new tool for public health surveillance. Injury prevention. 2006; 12(2):3–5. doi: 10.1136/ip.2006.012518 17170168 PMC2563479

[46] Chiang HC, Chen HS, Tai CW, Lee MB. National suicide surveillance system: experience in Taiwan In: HEALTHCOM 8th International Conference on e-Health Networking, Applications and Services, New Delhi, India. 2006; pp 160–164. 10.1109/HEALTH.2006.246439.

[47] Paulozzi LJ, Mercy J, Frazier L, Annest JL, Centers for Disease Control and Preventionet. CDC’s National violent death reporting system: background and methodology. Injury prevention. 2004; 10(1):47–52. 10.1136/ip.2003.003434. 14760027 PMC1756538

[48] Ward E, Arscott-Mills S, Gordon G, Ashley D, McCartney T, Jamaican Injury Surveillance System. The establishment of a Jamaican all-injury surveillance system. Int J Inj Contr Saf Promot. 2002; 9(4):219–225. 10.1076/icsp.9.4.219.13677. 12613100

[49] Arscott-Mills S, Holder Y, Gordon G, Jamaican Injury Surveillance System. Comparative evaluation of different modes of a national accident and emergency department-based injury surveillance system: Jamaican experience. Injury control and safety promotion. 2002; 9(4):235–239. 10.1076/icsp.9.4.235.13683. 12613102

[50] Brazil. Manual de procedimento do sistema de informações sobre mortalidade. Brasília, Brazil. 2001. https://bvsms.saude.gov.br/bvs/publicacoes/sis_mortalidade.pdf.

[51] Butchart A, Peden M, Matzopoulos R, Phillips R, Burrows S, Bhagwandin N, et al. The South African national non-natural mortality surveillance system—rationale, pilot results and evaluation. S Afr Med J. 2001; 91(5):408–417. 11455806

[52] Mackenzie SG, Pless IB. CHIRPP: Canada’s principal injury surveillance program. Canadian hospitals injury reporting and prevention program. Injury prevention. 1999; 5(3):208–213. doi: 10.1136/ip.5.3.208 10518269 PMC1730529

[53] Birkhead GS, Galvin VG, Meehan PJ, O’Carroll PW, Mercy JA. The emergency department in surveillance of attempted suicide: findings and methodologic considerations. Public Health Rep. 1993; 108(3):323–331. 8497570 PMC1403383

[54] Silverman MM, De Leo D. Why there is a need for an international nomenclature and classification system for suicide. Crisis. 2016; 37(2):83–87. doi: 10.1027/0227-5910/a000419 27232426

[55] Sara GE, Wu J. Enhanced self-harm presentation reporting using additional ICD-10 codes and free text in NSW emergency departments. Public Health Res Pract. 2023; 33(3):33012303. doi: 10.17061/phrp33012303 36792352

[56] Simon GE, Shortreed SM, Boggs JM, Clarke GN, Rossom RC, Richards JE, et al. Accuracy of ICD-10-CM encounter diagnoses from health records for identifying self-harm events. J Am Med Inform Assoc. 2022; 29(12):2023–2031. doi: 10.1093/jamia/ocac144 36018725 PMC9667165

[57] Walker S, Chen L, Madden R. Deaths due to suicide: the effects of certification and coding practices in Australia. Aust N Z J Public Health. 2008; 32(2):126–130. doi: 10.1111/j.1753-6405.2008.00187.x 18412681

[58] Ochuku B. K., Johnson N. E., Osborn T. L., Wasanga C. M., & Ndetei D. M. (2022). Centering decriminalization of suicide in low—and middle—income countries on effective suicide prevention strategies. Frontiers in psychiatry, 13, 1034206. doi: 10.3389/fpsyt.2022.1034206 36465309 PMC9712720

