# Supplementary material for: Characteristics of surveillance systems for suicide and self-harm: A scoping review
Source: PLOS Glob Public Health. 2024 Jul 2;4(7):e0003292. doi: 10.1371/journal.pgph.0003292 (PMC11218961; doi:10.1371/journal.pgph.0003292)
Supplement: S1 Table — (DOCX) [file pgph.0003292.s001.docx]

**SUPPLEMENTARY MATERIALS**

**S1 Table.** Search strategy based on the mnemonic PCC (mnemonic for Population, Concept and Context)

| **Review question: What are the characteristics of surveillance systems for self-harm and suicide?** | | |
| --- | --- | --- |
| Population | Public Health Surveillance: “the ongoing, systematic collection, analysis, and interpretation of health-related data with the purpose of preventing or controlling disease or injury, or of identifying unusual events of public health importance, followed by the dissemination and use of information for public health action” (MeSH term) | “surveillance systems” OR “surveillance system” OR “public health surveillance” OR “population health surveillance” |
| Concept | Suicide: “the act of killing oneself” (MeSH term)  Self-harm: “any act of self-poisoning or self-injury carried out by an individual irrespective of motivation” (NICE, 2012; WHO, 2016) | “self-injurious behavior” OR “self injurious behavior” OR “self-injurious behaviors” OR suicide OR "suicide attempt" OR "suicide completed" OR "self-harm" OR “self harm” OR “nonsuicidal self injury” OR "nonsuicidal self-injury" OR “nonsuicidal self injuries“ OR “non-suicidal self injuries” |
| Context | Considering the review aims, no context limitation will be considered. | - |
