# Supplementary material for: Characteristics of surveillance systems for suicide and self-harm: A scoping review
Source: PLOS Glob Public Health. 2024 Jul 2;4(7):e0003292. doi: 10.1371/journal.pgph.0003292 (PMC11218961; doi:10.1371/journal.pgph.0003292)
Supplement: S2 Table — (DOCX) [file pgph.0003292.s002.docx]

**S2 Table:** Synthesis of the characteristics of general surveillance systems (includes suicide and self-harm).

| **Name of system**  **(Country)** | **Level of source** | **Data source** | **System description**  **(Category, data collection and analysis and dissemination)** |
| --- | --- | --- | --- |
| Observatorio Regional de Salud Mental - SICA  (Central America and Dominican Republic) | International | National and Department of Health data coming from different surveillance systems across eight countries members of the SICA (Belize, Costa Rica, El Salvador, Guatemala, Honduras, Nicaragua, Panama, and Dominican Republic) | Observatory collecting data regarding age, gender, time and date, place and method used for the attempt. |
| Sistema Nacional de Vigilancia Epidemiologica - SINAVE  (Guatemala) | National | Surveillance form used for suicide behaviour | System. Register performed by services from primary, secondary, and tertiary levels monthly. Data is sent to the *Direccion de Area de Salud* (national epidemiology level) to be analysed and orient decision-making on SIGSA (generating policy). |
| Sistema de Vigilancia de Lesiones - SIVILE  (Argentina) | Nacional | Register form containing info from interviews with the victim and/or family/friends about the incident, administrative sources, clinical history, and interview with the doctor. Unique form for intoxications, injury and suicide | System collecting general and specific data regarding the event, sociodemographic information, injury characteristics, exam results and health appointment observations; Data collection: physical form filled in by the professionals in the service. The data is then transferred to the online system. |
| Injury Surveillance System (Iran) | National and Provincial | All admission to A&E due to injuries | The system collects data about name, age, sex, location (urban or rural area), injury, date and location, injury type and outcome. Data on all injuries in emergency rooms following admission (all health care institutions at different levels). The data flow from peripheral health centres to provincial levels and the MOHME (Ministry of Health and Medical Education) for compilation, analysis, interpretation, reporting, dissemination, and follow-up activities. The data received in the centre will be analysed at a one-year periodical interval, mainly in descriptive format and then summarised as brief reports. |
| Notifiable Diseases Information System - SINAN (Brazil) | National | Form for registering interpersonal and self-inflicted violence. The form is standardized and pre-numbered by the Department of Health | System. General data, individual notification, residence information, information about the victim, event data, type of violence, sexual violence, info about the suspect, referral and outcome data, complementary information and clinical observations. The form is filled in by the healthcare services (it can also be filled in at other services such as schools, social services, etc.). The form is typed into the system by local health or epidemiological teams. Professionals are required to fill in the form up to 24 hours of contact with the case. This data is sent to local epidemiological services weekly. |
| Mortality Information System - SIM (Brazil) | National | Death certificates (DC) | System. Data regards identification, residence, place of occurrence, condition and causes of death, doctor registering and type of external cause; The register is under a medical professional responsibility. The DC is printed and pre-numbered in a three-copy form by the Department of Health (one goes to the local health secretary, to the deceased family and one is kept in the unit responsible for the register). Local health departments collect the forms in all health units. After processed, revised, and corrected data is typed in the online regional database by the regional health department. The data then goes to CGAIS, who analyses and includes every data in the national database. |
| Surveillance System for Violence and Accidents - VIVA (Brazil) | National | Form for registering interpersonal and self-inflicted violence (Sinan) and Form for violence and accidents in emergency services (Epi Info) | Observatory with 2 components (I – Continuous surveillance - Viva Contínuo/Sinan, data about interpersonal and self-inflicted violence and II – Sentinel surveillance - Viva Inquérito, survey in emergency services, during 30 consecutive days. Data collected every 3 years; |
| National Ambulance Surveillance System - NASS (Australia) | National | Elletronic patient care records (ePCR) | System collecting demographic data, occurrence location, clinical information, treatment, type of violence, intention, method, mental health disorders, alcohol and drugs use, risk factors and textual description of the paramedics’ clinical evaluation. Ambulance calls for self-harm are filtered and classified considering the presence of self-harm before or during the appointment. Four categories are defined and coded in a local level (ambulance services): self-injury, suicidal ideation, suicide attempt, and suicide. After coding and revision at the local level, data is sent to the Turning Point (a national centre for research and treatment of addictions). |
| National Non-Natural Mortality Surveillance System - NMSS  (South Africa) | National | Register (physical form) of non-natural deaths known or suspected after medico-legal investigations (mortuary, lab exams, court investigations and police information). | System collecting 23 variables – personal and demographic data, conditions of death and data about the event. Data is collected by a mortuary professional responding to the call. The case is registered in a 2-copy paper form and typed into the system. One of the copies goes to the court. Data is collected weekly, monthly or fortnightly. After collected, data is integrated and analysed in the NMSS integrator sector of data by specialised researchers. These coordinating centres are responsible for developing general and specific reports. |
| National Electronic Injury Surveillance System - NEISS-AIP (United States) | National | Cases from the emergency services systems. | System. Collect additional data about Self-directed violence such as risk factors and substance use that are not available in any other databases. The data entry is performed by 1-2 coders per hospital. |
| National Violent Death Reporting System – NVDRS (United States) | National (state-based) | Death certificates, medical examiner/coroner records, law enforcement records and crime laboratory records. | System collecting sociodemographic characteristics of deceased persons and basic demographic characteristics of both alive and deceased suspects. Information on circumstances contributing to the deaths, such as (for suicides and deaths of undetermined intent) circumstances related to mental health, disclosed intent, and precipitating factors; and felony related or non-felony related circumstances for homicides. Data on interpersonal relationships (for example, the suspect was the victim’s spouse), victim toxicology (for example, the victim’s blood alcohol content), and the mechanisms of injury (for example, blunt instrument, poisoning, etc). Data analysis is performed according to the availability of the Death Certificates. Trained professionals are responsible to add data to the system. Trained coders will code the deaths using standard definitions. Data is added to the system as soon as they are available (usually on a monthly basis – this can vary from two months to 2 years when it comes to legal reports). Data is sent to the software during night and CDC gives feedback to the member states regarding the quality of their data. States are encouraged to build up its own protocols for data quality. |
| Jamaica Injury Surveillance System – JISS (Jamaica) | Regional -  5 emergency departments | Hospital triage form | System collecting qualitative survey regarding the injury – case identification depends on the existence of service triage. Answers are inserted immediately in the computer-based system by the professional responsible for the register. There is also a confirmation codding inserted in the system for each patient attended in the A&E. Data collection is performed in 24 hours by clerk staff on shift. Data is published monthly in reports made by hospital and regional professionals. This generates data to plan and evaluate care protocols. |
| Canadian Hospitals Injury Reporting and Prevention Program - CHIRPP (Canada) | Paediatric and general hospitals | Questionnaire about the injury circumstances | Program. Collect data about victim’s (such as date of birth, sex, idiom and residence), injury circumstances (location, area, context, breakdown, mechanism or contributing factors intention), also descriptive variables such as how and where the injury occurred as well as products involved. An administrative clerk provides the paper form to parents. Medical notes are included in the back of the questionnaire. Data is gathered monthly. Emergency rooms send data to the program’s centre in Ottawa. In the centre, a team of trained professionals is responsible for entry and treatment of the data in the main database. |
| *The Accident and Emergency Statistical Report - A&ESR (Jamaica) | National | A&E admissions (chart containing type by age and sex) | System; The system includes data on intentional and unintentional injuries, respiratory tract infections, gastroenteritis and other presenting conditions. A team of trained medical staff retrospectively fill in a chart with each patient’s visit to the emergency department (primary diagnosis categorised by age and sex). The register is completed daily in the hospitals in order to create a weekly and eventually monthly log. The hospitals send its data monthly to the Health Information Unit of the Department of Health as part of the Hospital Monthly Statistics Report. |
| *Patient Administration System /Jamaica Injury Surveillance System - PAS/JISS (Jamaica) | National | Clinical appointment (from five participant hospitals) | System. Personal and clinical data of victims presenting to the A&E because of events that caused injury. A case is defined as the first presentation/visit occasioned by a potential injury event, be it intentional or non-intentional. |

*Systems presented in the same included material.
