# Supplementary material for: Characteristics of surveillance systems for suicide and self-harm: A scoping review
Source: PLOS Glob Public Health. 2024 Jul 2;4(7):e0003292. doi: 10.1371/journal.pgph.0003292 (PMC11218961; doi:10.1371/journal.pgph.0003292)
Supplement: S3 Table — (DOCX) [file pgph.0003292.s003.docx]

**S3 Table:** Synthesis of the characteristics of specific surveillance systems for suicide and self-harm.

| **Name/**  **Location** | **Level of source** | **Data source** | **System description**  **(Category, collected data, data collection and analysis and dissemination)** |
| --- | --- | --- | --- |
| Sistema Nacional de Vigilancia en Salud Pública -SIVIGILA  (Colômbia) | National | Notification forms for basic and supplementary data (associated factors, psychiatric disorders, mechanism, method, risk assessment). | Category: System; Data collected; Data collection and analysis: in tables by age, life cycle, gender, and place of residence. The data collection is ongoing with weekly collection and the possibility of adjustments within the epidemiological period. Frequency: Within the next 24 hours after the information about them has been recorded. Dissemination: quarterly reports. Data used for prevention in individual actions (intervention and follow-up) and collective actions (strengthening coordination, awareness, social education, early identification, and case referral). |
| National Suicide Surveillance System - NSSS (Taiwan) | National | Integration of data from hospital reports, fire departments, social services, education, and the labor department. | Category: System; Data collected: Not specified; Data collection and analysis: In its new version, data collection is carried out through reporting units (health services, police, fire departments, civil affairs, education, social welfare, and labor departments). These data are sent to the municipal health department and to specialised services in the area of the person who receives home visits and telephone contacts. If necessary, cases are referred to relevant services. Frequency: Within 24 hours. Dissemination: All reports are integrated into the server of the Suicide Prevention Surveillance System of the Department of Health (of which NSSS is a part). This server receives the data and transfers it to the E-service. Local servers only have access to the data of victims within their jurisdiction. |
| *Coronial Suspected Suicide Data Sharing Service – CDS (Nova Zelandia) | National | Police records of suspicious deaths by suicide. | Category: System; Data collected: Not specified. Data collection and analysis: Deaths are also reviewed by the Suicide Mortality Review Committee. Statistical analysis. Frequency: Within 6–12 h after a death was first attended by Police. Dissemination: District notification of suspected suicides, through encrypted email, for postvention actions. General data is reported annually by the Coroner’s office. |
| Suicide Prevention Applications Network (SPAN) | National | Routine reports of suicide events. | Category: System; Data collected: date, circumstances, method, health information; Data collection and analysis: Suicidal behavior reports can be completed for events treated in hospital and outpatient settings and for events that suicide prevention coordinators have knowledge of but were not treated at a Veterans Health Administration (VHA) medical center. Frequency: Not specified. Dissemination: Suicide prevention coordinators submit these reports to a centralized database that forms part of an internal resource known as the Suicide Prevention Applications Network. Clinically, the completion of an SBR and the entry of a suicide event into the Suicide Prevention Applications Network indicate suicide risk and result in: Placement on the Veterans Health Administration's high-risk list. Patient's medical record in the Veterans Health Administration being marked with a treatment flag. Improvements in care and case management. |
| Police-led RTSS system  (Grã-Bretanha) | National | Police records of suspicious deaths by suicide. | Category: System; Data collected: demographic information, such as age, gender, and nationality, as well as life events, mental health problems, and previous police contact. The police also record the details of the circumstances of the death, such as the time, date, location, and method. Data collection and analysis: Each police force has a standardised recording template for cases in its jurisdiction. The records are sent to the BTP central via secure email. Frequency: Monthly. Dissemination: The data sent to the BTP is compiled and analyzed, allowing for the creation of a national monitoring system for suspected suicide deaths. |
| Subsistema de Vigilancia epidemiológica de la conducta suicida -SISVECOS (Colombia) | National | Notification Form | Category: System; Data collected: Face A - Basic Patient Data (victim identification); Face B - Supplementary Data (Companion's Name and Phone Number, Socioeconomic Status, Sexual Orientation, Differential Populations, Detected Case, substance use, Type of Behavior, Date of Occurrence, Time of Occurrence, Occurrence Setting, Mechanism, Associated Mental or Behavioral Disorder, Person Referred to Mental Health Services), and Face C - Case Follow-up (Date of First Contact, SISVECOS Intervention Start Date, Reported As, Family Composition, Family Type, Family Life Cycle, Family Apgar Score, Extended Family, Recreation, Education, Work, Health, Religious Groups, Neighbors, Community, Sports, Partner, Friends, Risk Assessment Score, Primary Triggering Event, Secondary Triggering Event, Tertiary Triggering Event, Recurrent Suicidal Behavior, Number of Recurrences, Classification of Suicidal Behavior Following Follow-up, Was the Case Handled by DUES (CRUE). Data collection and analysis: Notification forms should be completed within 24 hours after the case. Frequency: Not specified. Dissemination: Not specified. |
| *Interim Queensland Suicide Register (iQSR) (Queensland, Australia) | State-based | Provide real-time information about suspected suicides. | Category: System. Data collected: Demographic items, “motives/triggers” for suicide, next-of-kin details (dependent on consent to contact for research purposes), circumstances of death, internet use, communications of intent, prior suicide attempts, and incident summaries. Data collection and analysis: Suspected cases are defined as 'possible,' 'probable,' or 'beyond reasonable doubt’. Those in the latter two categories are considered “suspected suicides.” Cases are reviewed when the coronial investigation is completed. Frequency: Triweekly updates of deaths that have occurred within this timeframe. Dissemination: Fortnightly updates for the funding agency and quarterly reports for the mental health organisations. Annual reports are released publicly. |
| *Victorian Suicide Register – VSR (Victoria, Australia) | State-based | Police-led real-time suicide surveillance system. | Category: System; Data collected: Police death report, medical and forensic post-mortem reports (autopsy and toxicology) and coroner's summary (health records, statements from family members, witnesses, photographs, maps). Data collection and analysis: Cases of probable suicide are classified as “intentional self-harm” and cases of possible suicide are classified as “unable to be determined” while the coronial investigation is underway. The cases undergo a thorough review in the final stage of the coronial investigation. Frequency: Updated each weekday and an enhanced dataset to record more in-depth data following the conclusion of coronial investigations. Dissemination: Real-time data is disseminated to Victorian and Commonwealth health authorities. Monthly update reports on suicides are published publicly. Data access for other purposes is contingent on approval from the Coroners Court of Victoria. Use data for informs local suicide prevention planning, vulnerability of at-risk individuals, identifying priorities for coronial investigation, educating the public about suicide, monitoring suicide trends and alerting local health services to potential emerging issues. |
| *Thames Valley Police Real-Time Suicide Surveillance - TV-RT-SSS (Buckinghamshire,  Berkshire, and  Oxfordshire, England) | Regional | Police-led real-time suicide surveillance system. | Category: System. Data collected: The TV-RT-SSS contains 11 variables: the deceased’s demographic information, possible triggers, mental health history, health service use, and circumstances of death. Data collection and analysis: Data initially relates to cases of “suspected suicide” as identified by attending police officers or suspected by the coroner’s team. Then later confirmed and validated with coroner records following inquest outcomes. Frequency: Within 24–72 h after a death has occurred. Dissemination: Information is shared with relevant partners in aggregated form to inform service responses to emerging suicide links, identify clusters, increase capacity for early intervention, assist with optimizing resource allocation and facilitate implementation or activation of local plans. |
| *Suicide and Self-Harm Observatory - SSHO  (County Cork, Ireland) | Regional | Real-time data on suspected suicides. | Category: Observatory. Data collected: Database comprising 16 variables that capture demographic information related to the deceased, circumstances of death, history of abuse, and utilisation of mental health services. Data collection and analysis: Data related to instances of “suspected suicide,” classified by the coroner based on evidence from various sources, including the police, witnesses, and family accounts. Cases are reviewed once the inquest has concluded. Frequency: Fortnightly updates of deaths that have occurred within this timeframe. Dissemination: Information is shared with the health organizations and suicide prevention entities to inform bereavement support and community prevention activities. Aggregated information is periodically shared with key stakeholders involved in suicide prevention plans in the region on a need-toknow basis. |
| Suicide Prevention and Implementation Research IniTiative - SPIRIT (India) | National | Data from public and private healthcare services, police records, as well as semi-structured interviews with informants. | Category: Program; Data collection and analysis: Healthcare data was collected quarterly from healthcare systems; police data was collected annually from police systems, and community data was collected from key informants (cases that occurred within the 3 months of collection or the previous month). Frequency: Not specified. Dissemination: Not specified. |
| Emergency department surveillance system-case  (Atlanta, GA) | Regional | Ambulance service records | Category: Sistema; data collected: Mode of arrival, Mental Status, Nature of Injury, ED Discharge Diagnosis, Concurrent Drug Use, Evidence of intent to commit suicide, Disposition (Admitted to this hospital, Transferred to another facility, Discharged home, Died in ED), Degree of certainty that suicide attempt occurred, Was the attempt life-threatening, Have you ever received counseling in the past) Nature of injury categories were taken from the International Classification of Diseases coding scheme. |
| Apache Surveillance System (EUA) | Local | Directly with informants (who witnessed or were familiar with the case) through a physical form. | Category: System; Data collected: The collected data include demographic information, behaviour details, and case follow-up. Data collection and analysis: Cases are reported by individuals who have had contact with the case (e.g., school, social services, family, etc.) - most are reported in real-time, but some are reported well after the event. This communication with Celebrating Life is done via fax, paper, or telephone. After receiving the case, Celebrating Life conducts follow-up contact to gather more information within 24 hours. Frequency: Data is analysed quarterly, semi-annually, and annually and reported to the WMAT council (municipality), the local hospital's clinical director, and the local hospital's preventive medicine epidemiologist. Rates and trends are analysed annually and shared with local leaders for early identification of risk factors and intervention possibilities. Dissemination: Data is analysed and coded, and individuals are referred to the Apache Behavioral Health Services (local community mental health center). |
| Self-Harm Surveillance Register (Inglaterra) | Local | Integrate data from the patient administration, mental health patient record system, risk assessment form from the EU department, psychosocial assessment form, and local coroner's data. | Category: System; Data collected: Sociodemographic data and detailed information on self-harm episodes, including patient demographics, clinical characteristics, methods of self-harm, previous history of self-harm, mental health diagnoses, and psychosocial assessments. Data collection and analysis: The record collects and compares data almost simultaneously and in more detail than routine hospital information systems. It allows for the assessment of changes in the incidence of self-harm, the impact of service changes, and patient management. Frequency: Not specified. Dissemination: Not specified. |
| Celebrating Life Suicide Surveillance and Case Management System (CL)  (EUA) | Local | Admission form (age, gender, residence, marital status, type, event location, and reporter's name) | The tribal law requires registration of all suicide-related events. Admission form (age, gender, residence, marital status, type, event location, and reporter's name). People report cases to the Community Mental Health Workers. The cases are managed by the Apache team and referred to a designated case manager who assesses the risk and proposes follow-up (30-90 days). |

*As systems are presented in a single material that analyzed five suicide surveillance systems [26].
